# The Question of Mass Contributions

**Published:** 1921-07-16

**Authors:** 


### THE QUESTION OF MASS CONTRIBUTIONS.

A Conference of representatives of London Hospitals, called at the instance of King Edward's Hospital Fund, was held at the Mansion House on Wednesday, July 6, Lord Stuart of Wortley, Chairman of the King's Fund Policy Committee, presiding.

Invitations were issued to 112 hospitals, and 161 persons were present, of whom 154 represented 94 hospitals, viz.: All the larger general hospitals with medical schools, excepting Charing Cross Hospital. *Larger General without Medical School*: The Dreadnought, German, Great Northern, London Homœopathic, Metropolitan, Prince of Wales', and West London. *Smaller General*: Bolingbroke, French, Hampstead, Italian, King Edward Memorial, London Temperance, Mildmay Memorial, Mildmay Mission, Miller, Poplar, Royal (Richmond), St. John's (Lewisham). *Chest*: City of London Chest, Consumption, Royal Chest. *Women's*: Chelsea, Grosvenor, Samaritan Free, South London, Stoke Newington Home Hospital. *Children*: Belgrave, East London, Evelina, Great Ormond Street, Paddington Green, Queen's, Roll of Honour, Victoria. *Ophthalmic*: Central London, Royal Eye, Royal London, Royal Westminster. *Epilepsy*: Maida Vale, National, West End. Nine cottage hospitals, seven lying-in hospitals, and twenty-three other hospitals.

The object of the Conference was to discuss the three schemes of "mass contributions" referred to by Lord Cave's Committee in their final Report as likely to provide the key to the problem of hospital finance—viz.: (*a*) organised weekly contributions of wage-earners on a large scale, but without any definite insurance system; (*b*) the "Oxford" scheme, under which the contributors pay 2d. per week, and are entitled to free treatment; whereas non-contributors are expected to pay towards their maintenance; (*c*) the "Sussex" scheme, an insurance system for hospital and other benefits under which, in the modified form proposed for London, the contributor pays 5d. per week as premium and the hospital gets about as much as under the "Oxford" scheme, the premium also providing the members with other facilities, such as private consultations, dental treatment, etc.

### No Time to Lose.

Lord Stuart of Wortley impressed on the meeting that the time allowed for the re-establishment of the finances of the hospitals is very short. Lord Cave's Committee gave two years, but that presupposed the acceptance of its recommendations of a Government emergency grant of £1,000,000 for the first year, and possibly less in the second, whereas the Government has only suggested £500,000 for the first year. Emergency assistance is conditional on the hospitals taking for themselves all possible steps to get over their financial troubles. This needs not only reduction of expenditure but also increases in income, particularly by what Lord Cave's Committee called "moderate and continuous contributions from all classes." After describing the three schemes mentioned in Lord Cave's Report, Lord Stuart pointed out that the King's Fund has not pronounced in favour of any particular method, the choice of which must arise from experience sought by the hospitals themselves; nor do they think it necessary that the same method should be adopted by all the hospitals. Three of the large hospitals had that morning announced a decision to co-operate in giving the "Sussex" scheme a trial. The King's Fund would welcome any practical experiments based on the findings of Lord Cave's Committee.

### Views of Individual Hospitals.

Sir William Lawrence (St. Bartholomew's Hospital) said that St. Bartholomew's Hospital would be willing to try Scheme A if it did not interfere with receipts from patients' payments. Scheme C implies some form of contract, whereas now the passport of admission is to be one of the sick poor. Sir James Harrison (Royal Eye Hospital) suggested that Scheme A could be organised by the Saturday Fund and the League of Mercy. Mr. Stuart de la Rue (Great Northern Hospital and Royal Chest Hospital) thinks that in any scheme some form of contract is inevitable. The question of areas and methods of collection would need consideration. Lord Goschen (Guy's) advocated co-operation between the Saturday Fund and the hospitals, singly or in groups. The Sussex scheme means raising the income limit, which now confines treatment to the sick poor. Sir Edward Penton (Middlesex Hospital) suggested making use of municipal patriotism. The voluntary hospitals are popular with the workers, if they are approached direct, as is done by the Saturday Fund. There should be a central advisory committee to help the King's Fund to arrange the work in districts. Lord Hambleden (King's College Hospital) referred to the difficulty that in London men work in one district and live in another. The schemes must, therefore, cover all London, but need not be all of the same type. The areas might sometimes be municipal and sometimes centre round a large hospital. Sir John Young (Central London Throat) recommended central machinery for co-ordination and delimitation of areas. Mr. Meller (Queen's Hospital for Children) urged that the appeal should not be confined to wage-earners, as they already contribute largely, and this point was later emphasised by Mr. T. Glenton Kerr. Mr. Austin Taylor (Westminster Hospital) suggested co-operation with other hospitals in the Westminster areas. He asked whether the three hospitals which were trying the Sussex scheme would be observing any such limit of area. Mr. Radford (Woolwich and District War Memorial Hospital) said that it is essential to give the wage-earners representation on the local organising committees, on the central committee, and on the hospital committees. Sir Charles Stone (Miller Hospital) asked how a system of pooling wage-earners' contributions would affect a hospital situated in a factory district.

### Details of a Concrete Scheme Asked For.

Mr. Morris (London Hospital) submitted that the rise in cost justified a rise in the income limit, and that the Sussex scheme would, by means of insurance during health, provide the black-coated poor with facilities now only open to the very rich or the very poor. The difficulty of localising appeals is that supporters of hospitals, especially in poor districts, often live at a distance. The Sussex scheme, however, is being organised by a committee, the three hospitals co-operating by providing treatment. Sir Alan Anderson (King's Fund) pointed out that the different schemes are not hostile. Some development on the lines of Scheme A seems inevitable for the support of all hospitals; Schemes B and C both involve a contract; Scheme C is not incompatible with the other schemes, and the action of the three hospitals in giving it a full trial is in accordance with the suggestion of Lord Cave's Committee. Mr. Ferard (Victoria Hospital for Children) hoped that if a committee is appointed it will work out a concrete scheme showing how London can be divided into districts, how individual hospitals should appeal, so as not to lose their individuality, and how the money will be distributed. Lord Arran (Royal Westminster Ophthalmic Hospital) said that the case of special hospitals needs attention, as they have no definite area but deal with people all over London.

Lord Stuart of Wortley pointed out that Lord Cave's Report specifically referred to contributions from "all classes," and that it recommended representation on hospital committees as essential to the success of organised collections from wage-earners.

On the motion of Lord Hambleden, seconded by Sir Edward Penton, it was unanimously agreed "That this Conference suggests that King Edward's Hospital Fund should co-operate with the London Regional Committee of the British Hospitals Association, the Hospital Sunday Fund, the Hospital Saturday Fund, and the League of Mercy, in the organisation of local collections from employees throughout the London area."

The proceedings terminated with a vote of thanks to the Lord Mayor for providing the room at the Mansion House.

